# Chernobyl Birds Have Smaller Brains

**DOI:** 10.1371/journal.pone.0016862

**Published:** 2011-02-04

**Authors:** Anders Pape Møller, Andea Bonisoli-Alquati, Geir Rudolfsen, Timothy A. Mousseau

**Affiliations:** 1 Laboratoire d'Ecologie, Systématique et Evolution, CNRS UMR 8079, Université Paris-Sud, Orsay, France; 2 Department of Biological Sciences, University of South Carolina, Columbia, South Carolina, United States of America; 3 Norwegian Radiation Protection Authority (NRPA), Department of Environmental Radioactivity, The Polar Environmental Center, Tromsø, Norway; Freie Universitaet Berlin, Germany

## Abstract

**Background:**

Animals living in areas contaminated by radioactive material from Chernobyl suffer from increased oxidative stress and low levels of antioxidants. Therefore, normal development of the nervous system is jeopardized as reflected by high frequencies of developmental errors, reduced brain size and impaired cognitive abilities in humans. Alternatively, associations between psychological effects and radiation have been attributed to post-traumatic stress in humans.

**Methodology/Principal Finding:**

Here we used an extensive sample of 550 birds belonging to 48 species to test the prediction that even in the absence of post-traumatic stress, there is a negative association between relative brain size and level of background radiation. We found a negative association between brain size as reflected by external head volume and level of background radiation, independent of structural body size and body mass. The observed reduction in brain size in relation to background radiation amounted to 5% across the range of almost a factor 5,000 in radiation level. Species differed significantly in reduction in brain size with increasing background radiation, and brain size was the only morphological character that showed a negative relationship with radiation. Brain size was significantly smaller in yearlings than in older individuals.

**Conclusions/Significance:**

Low dose radiation can have significant effects on normal brain development as reflected by brain size and therefore potentially cognitive ability. The fact that brain size was smaller in yearlings than in older individuals implies that there was significant directional selection on brain size with individuals with larger brains experiencing a viability advantage.

## Introduction

Impaired brain development is linked to oxidative stress because of the high lipid content of brains. Large-brained individuals must be capable of continuously supplying the brain with high levels of oxygen for neuronal ion pumping, synthesis of neurotransmitters and protection from toxic compounds (e. g. [1]–[3]). This makes brain maintenance a highly oxidizing process that requires large amounts of antioxidants, in particular glutathione. Therefore, any environment with low antioxidant levels and/or high rates of use of antioxidants will provide a challenge to normal brain development. One such extreme environment is Chernobyl because high levels of background radiation increase oxidative stress [4], cause high rates of use of antioxidants, and hence reduce levels of circulating and stored antioxidants [5]–[14].

Evidence for developmental errors in the nervous systems of people exposed to radiation is widespread (e. g. [15]–[17]), including reduced head size (e. g. [18], [19]) and brain damage (e. g. [20]). Low levels of ionizing radiation cause changes in both central and autonomous nervous systems and can cause radiogenic encephalopathy [21]. Electroencephalographic studies revealed changes in brain structure and cognitive disorders [22]. Indeed Yablokov et al. [23] summarized an extensive literature on the effects of radiation on cognitive performance as a consequence of the Chernobyl disaster. However, psychological effects of radiation from Chernobyl have recently been attributed to post-traumatic stress rather than developmental errors (e. g. [24]), and increased levels of neural tube defects in contaminated areas may be ascribed to low-dose radiation, folate deficiencies or prenatal alcohol teratogenesis [17]. Surprisingly, studies of high school performance and cognitive abilities among children from contaminated areas in Scandinavia that were *in utero* during the Chernobyl disaster show reductions in high school attendance, have lower exam results and reduced IQ scores compared to control groups (e. g. [25], [26]). These cognitive effects are assumed to be due to developmental errors in neural tissue caused by radiation during early pregnancy. These differences in Scandinavia cannot readily be attributed to changes in social conditions during recent decades. Such social changes have characterized the now independent countries formerly belonging to the Soviet Union, where negative effects of post-traumatic stress have been suggested to account for psychological problems among children living in contaminated areas near Chernobyl (e. g. [24]).

Here, we tested whether brain size was reduced in birds living in areas differing in background radiation level due to fallout from Chernobyl. A second objective was to test whether brain size increased with age, as expected if there is viability selection against reduced brain size. The key advantage of this study stems from the fact that any observed differences in brain mass in birds associated with radiation cannot be attributed to post-traumatic stress as suggested for humans.

## Methods

### Study sites

**Figure 1:**
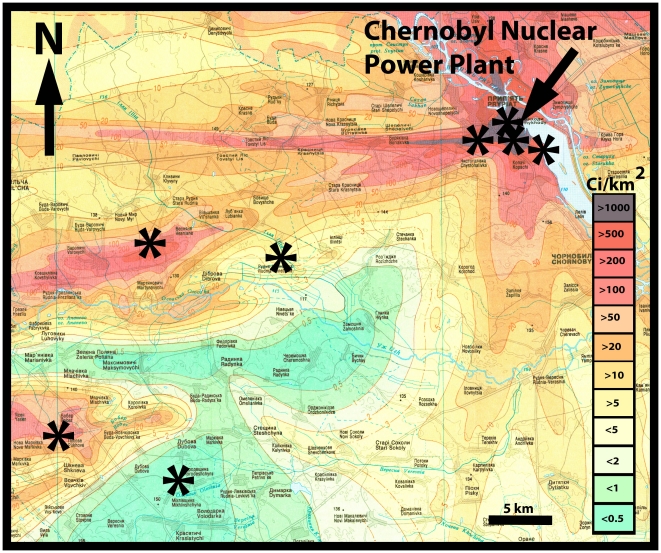
Background contamination levels (Ci/km^2^) in the Chernobyl region and location of study sites. Adapted from Shestopalov [28].

We captured 546 birds using 35 12 m mist nets in woodland that exhibit severe reductions in species richness and density of invertebrates and vertebrates [27] in eight different sites around Chernobyl, Ukraine (Fig. 1) during 25 May – 5 June 2010. 35 mist nets was the maximum that we were able to monitor in the areas with highest density. Each site was used for capture on two consecutive days thus ensuring a similar capture effort in all sample sites. Because the density of birds has been found to decrease with increasing background radiation level [27], we expected to catch fewer individuals at sites with high level background radiation. In addition, we captured barn swallows at farms where we have followed the population since 1991 (e. g. [11]). Capture of birds was conducted under permission from the authorities of the Chernobyl Exclusion Zone. A list of species and number of individuals is reported in Electronic appendix S1 together with information on morphology, age and background radiation.

### Measurements

#### Background radiation levels

Radiation levels in the field were cross-validated with measurements by Ministry of Emergencies, Ukraine. We measured aband g radiation at ground level at each capture point using a hand-held dosimeter (Model: Inspector, SE International, Inc., Summertown, TN, USA). Our data were validated against data from the governmental measurements published by Shestopalov [28], estimated as the mid-point of the ranges published, with analyses showing high degree of consistency between methods [29]. Background radiation levels are strongly positively correlated with internal dose levels for individual birds [30].

#### Morphological measurements

Upon capture all adult birds were measured, recorded in similar, standardized ways by APM, while TAM wrote down all measurements. Here, we only used mean length of wing and outermost and central tail feathers measured with a ruler to the nearest mm, keel length and beak length, width and height, and tarsus length with a digital caliper to the nearest 0.01 mm and body mass to the nearest 0.1 g with a Pesola spring balance. Measurements have repeatabilities above 94% [31], [32].

#### Brain size and head size

APM recorded to the nearest 0.01 mm maximum head length from tip of the beak to back of the head, maximum head width at the widest point at the back of the head, and maximum head height from top of the head to bottom behind the jaw. Head volume was subsequently estimated using the equation for an ellipsoid. Although head volume is a highly reliable correlate of brain volume across birds [33], we still explicitly tested if head volume in our sample predicted brain mass. Repeatability of head volume of the same individuals on different days has been found to be very high [32].

We obtained information on brain mass from the literature [32], [34]–[36] for the analysis of whether head volume predicted brain mass.

#### Sexing and aging

Birds were sexed and aged according to criteria in Svensson [37], but not all species could be aged because reliable criteria for aging are absent.

Summary statistics for head volume, brain mass, body mass and age are reported in Table S1 and data on background radiation, body mass, beak length and head volume for individual birds are reported in Table S2.

### Statistical analyses

Head volume, other morphological characters and background radiation were log_10_-transformed before analyses. We explicitly tested whether head volume predicted brain mass using our estimates of head volume combined with estimates of brain mass as reported in the literature. We tested whether there were outliers in this relationship using Cook's D as a test statistic [38].

We modeled head volume by using species, background radiation level nested within species (to account for the fact that we were interested in differences in head volume among individuals within species in response to changes in level of background radiation for these individuals) and body mass and body size measurements as predictors. This analysis was restricted to the sample of species for which information for at least two individuals differing in radiation level were recorded because within species responses can only be investigated when information for at least two individuals is available. We developed best-fit general linear models, relying on the software JMP [38], using Akaike's information criterion (AIC_c_) for small sample sizes as an estimate of the improvement in fit for addition of variables [39]. A change in AIC_c_ by 2.0 units is considered significant [39], and we thus produced final models that fulfilled this requirement.

## Results

### Brain size and radiation

Head volume indeed predicted brain mass (Figure S1), with brain volume accounting for more than 90% of the variance in brain mass. None of the species were significant outliers as reflected by Cook's D, showing that the relationship between brain mass and body size was homogeneous for the species including the six non-Passeriformes.

**Figure 2:**
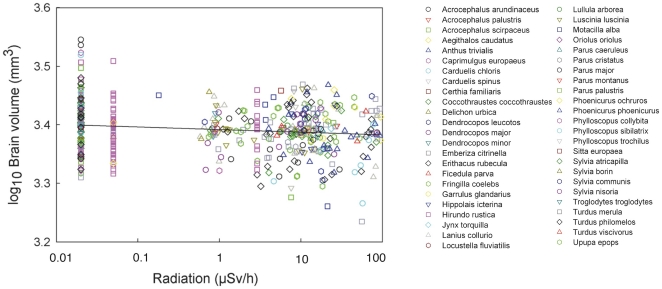
Head volume of birds (mm^3^) in relation to level of background radiation (µSv/h), after controlling for species and body mass. The line is the linear regression line with the equation log_10_(Head volume)  = 3.3918−0.0045 log_10_(Background radiation). Residuals from a model that included species as a predictor were added mean log_10_-transformed head volume 3.3934 to facilitate interpretation.

**Figure 3:**
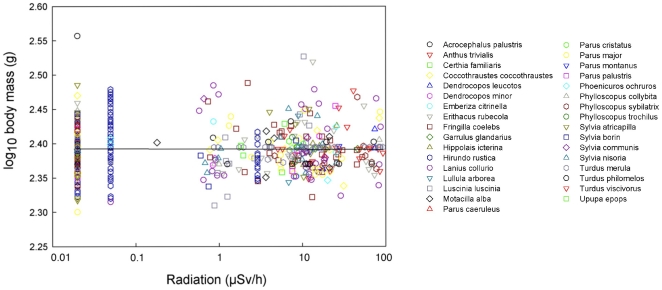
Body mass of birds (g) in relation to level of background radiation (µSv/h), after controlling for species. The line is the linear regression line with the equation log_10_(Head volume)  = 2.3922−0.0004 log_10_(Background radiation). Residuals from a model that included species as a predictor were added mean log_10_-transformed body mass 2.3922 to facilitate interpretation.

**Figure 4:**
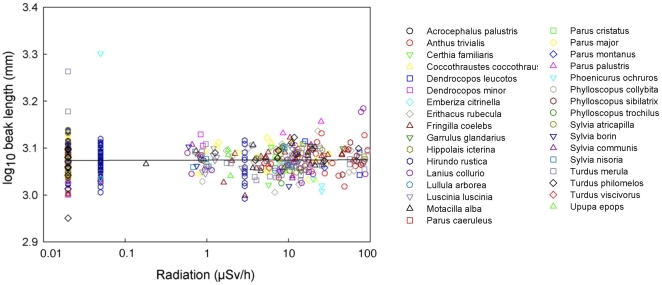
Beak length of birds (mm) in relation to level of background radiation (µSv/h), after controlling for species. The line is the linear regression line with the equation log_10_(Head volume)  = 3.0742−0.0004 log_10_(Background radiation). Residuals from a model that included species as a predictor were added mean log_10_-transformed beak length 3.0742 to facilitate interpretation.

Head volume decreased significantly with increasing radiation level, varying among species and with respect to body mass and keel length (Table 1; Fig. 2). Background radiation level accounted for 12.6% of the variance [40]. Body mass added to this model had a Δ AIC_c_  = 4.09, while keel length that reflects structural body size accounted for Δ AIC_c_  = 2.56. Hence, both these morphological characters were retained in the final model. All the remaining morphological traits only had Δ AIC_c_ <0.5, and these factors were not retained in the final model presented in Table 1. Only head volume varied significantly with background radiation level (Table 1), while that was not the case for any of the other morphological characters (Table S3). After accounting for the effect of differences among species, there was a reduction in mean brain volume by 5% across background radiation levels ranging from 0.02 to 94.61 µSv/h at capture sites (Fig. 2). There were additional significant effects of both radiation (F_1,449_ = 2.05, P = 0.0007) and sex, with males having relatively larger brains than females (F_1,449_ = 10.35, P = 0.0014, LSM (SE) for males after accounting for species and radiation effects: 3.273 (0.053), females: 3.262 (0.053); back-transformed values for males: 1875 mm^3^ (1.1), females: 1828 mm^3^ (1.3)). Two examples of the non-significant relationship between residual variation in other morphological characters and radiation after accounting for species are shown for body mass and beak length in Figs. 3 and 4.

**Table 1:** Head volume in relation to species, background radiation and body mass.

	Sum of squares	df	F	P	Slope (SE)
Species	1.008	32	13.93	<0.0001	
Radiation [Species]	0.146	33	1.96	0.0015	
Body mass	0.011	1	4.94	0.027	0.140 (0.063)
Keel length	0.008	1	3.59	0.059	0.177 (0.094
Error	1.013	448			

The model had the statistics F_67,448_ = 171.15, r^2^ = 0.96, P<0.0001.

### Brain size and age

In the smaller sample of species that could be aged at capture due to availability of aging criteria, there were significant effects of both radiation (F_1,284_ = 2.66, P<0.0001) and age, with yearlings having smaller head volumes than older individuals (F_1,284_ = 9.92, P = 0.0018, LSM (SE) for yearlings after adjusting for species, radiation and sex effects: 3.395 (0.008), older individuals: 3.414 (0.008), back-transformed values: 2483 mm^3^ (1.0), older individuals: 2594 mm^3^ (1.0)). This amounts to a difference of 4.3%, after back-transformation of log-values.

## Discussion

Birds living in areas with high levels of background radiation around Chernobyl have smaller brains as reflected by head volume. This effect was specific for brain mass, and it was not confounded by differences in structural body size or body mass. There were significant differences in the relationship between brain mass and radiation among species, implying that some species were more susceptible to the negative effects of radiation than others. Brain size was significantly smaller in yearlings than in older individuals, implying directional selection against small brain size.

Overall brain size has important implications for cognitive ability (e. g. [41]), partly because many brain components are strongly correlated with overall brain size, especially the large parts of the brain that are involved in higher-order and multimodal integration [42], [43]. Here we reported the first field study showing changes in brain size in response to an environmental variable, variation in low dose radiation. We found that radiation accounted for 12.6% of the variance in head volume, which must be considered a large effect size for biology. Cohen [44] considered effect sizes accounting for 9% of the variance to be intermediate and 25% of the variance to be large. A meta-analysis of all meta-analyses in biology revealed mean effect sizes accounting for 5–7% of the variance [45]. We found a 5% reduction in head volume across a radiation gradients varying by almost a factor 5,000. The magnitude of this reduction must be compared to the priorities of developing individuals likely to sacrifice the brain as one of the last organs. For example, Battley et al. [46] have shown that migratory birds can reduce the size of organs considerably during long-distance migration, but that organs vary in their timing of use for flight, suggesting that migrants have very clear priorities when sacrificing tissue. Battley et al. [46] demonstrated that brains belong to the category of the last organs to be sacrificed when migrants metabolize tissue for energy. If even small differences in brain size matters, as indicated by Battley et al. [46], we should be able to document differences in cognitive performance linked to differences in brain size. Indeed, a recent field study of brain size (as reflected by head volume) in the barn swallow *Hirundo rustica* showed that individuals with larger brains had earlier arrival during spring migration from Southern Africa, lived in larger colonies, were more difficult to capture, even more difficult to re-capture and had higher survival prospects than small-brained conspecifics living in the same sites [32].

As a corollary of the high priority that birds give maintenance of brain size during extreme environmental conditions [46], we should expect that a reduction in brain size is associated with significant reductions in viability. Thus, if even small reductions in brain mass are costly, we should expect significant phenotypic selection on brain mass when individuals for environmental reasons develop small brains for environmental reasons. Indeed, there was a significant difference in brain size between yearlings and older individuals by 0.14 standard deviation units, consistent with strong directional viability selection for larger brain size. Given that the present study was based on individuals captured at the peak of breeding late May-early June, this cohort must have experienced significant selective mortality since the previous breeding season when the yearlings arose as zygotes and developing embryos, especially among migrants that have flown to the winter quarters and back again and suffered significant mortality by doing so [47]. Thus the findings on reduced brain mass related to increased background radiation reported here must be conservative. Because head volume is a fixed morphological structure with no change once development has terminated during or shortly after fledging, the difference in head volume between yearlings and older individuals cannot be ascribed to phenotypic plasticity. The relationship between brain mass and background radiation differed among species, with some showing negligible effects, while others showed strong negative relationships.

The present study is based on the assumptions that (1) radiation exposure during the incubation and the nestling period affects brain development, and that such young birds subsequently return to breed in contaminated areas when adults; (2) parental germline mutations affect brain development; or (3) maternal antioxidant transfer to eggs affect brain development [11]. We have no explicit information on natal dispersal of birds in Ukraine. Although the surroundings of Chernobyl appear to constitute sink populations for the barn swallow, significant numbers of breeding birds of local origin still return [48]. Likewise, high frequencies of partial albinism and other abnormalities in barn swallows [49] and other birds (in the present study 50 out of 546 individuals) demonstrate that birds that have been subject to radiation do return to the contaminated study area. Given that thousands of square kilometers are contaminated with radiation in Ukraine, Belarus and Russia [28], birds that typically have mean natal dispersal distances of 1–10 km [50] will still end up in contaminated areas after dispersal.

We have no explicit information on the underlying mechanisms responsible for the reduction in head volume. Because the brain is particularly vulnerable to oxidative stress due to its high lipid content, maintenance requires large amounts of antioxidants (e. g. [1]–[3]). Given that antioxidant levels in birds in Chernobyl are severely depleted (e. g. [11]–[12]), reduced brain size may arise as a consequence of this depletion. Alternatively, radiation may have produced developmental errors in the brain, as reported for humans (e. g. [15]–[17]). However, if developmental errors induced by radiation were the underlying cause for a reduction in brain size, we should also expect to see significant effects for other morphological characters, which was not the case. Yet another possibility is that reduced food availability caused by reduced abundance of invertebrate prey [51] could have negatively affected brain development. While this in theory is a possibility, we are unaware of any empirical studies in the field or the lab showing such effects of food availability on brain development. Field studies of great tits *Parus major* in Ukraine have shown no significant effect of radiation on viability of nestlings [12], suggesting that food availability is not significantly restricted in contaminated areas. Finally, the studies of changes in organ size in migrating birds by Battley et al. [46] suggest that brain size belongs to the category of organs that are the last to be sacrificed even under extreme environmental challenges.

In conclusion, birds of a large range of common species showed reduced brain size as reflected by head volume in heavily contaminated areas around Chernobyl, consistent with the hypothesis that radioactive contamination has significant negative effects on normal brain development, and that such effects in birds cannot be attributed to post-traumatic stress as done for humans.

## Supporting Information

Table S1 Species names, number of individuals captured, brain volume (mm^3^), brain mass (g), body mass (g), and age distribution (% yearlings) and range in radiation levels (μSv/h) where a species occurred. Species with missing information on percentage of yearlings could not be aged according to criteria compiled in ref. [37]. See Methods for sources.
(DOC)

Table S2 Data on background radiation (μSv/h), body mass (g), beak length (mm) and head volume (mm^3^) for birds from the Chernobyl region. See Methods for further details.
(DOC)

Table S3 Relationship between morphology and radiation nested within species and species and body mass. Denominator degrees of freedom vary because of missing values due to broken feathers.
(DOC)

Figure S1 Brain mass (g) in relation to head volume (mm^3^) in different species of birds. The line is the linear regression line based on log10-transformed variables with the equation log_10_(Brain mass (g))  =  −2.803 + 0.798 log_10_(Head volume (mm^3^)), F_1,38_  =  316.93, r^2^  =  0.91, P < 0.0001.
(TIFF)
