# Supplementary material for: Chernobyl Birds Have Smaller Brains
Source: PLoS One. 2011 Feb 4;6(2):e16862. doi: 10.1371/journal.pone.0016862 (PMC3033907; doi:10.1371/journal.pone.0016862)
Supplement: Table S1 — Species names, number of individuals captured, brain volume (mm3), brain mass (g), body mass (g), and age distribution (% yearlings) and range in radiation levels (μSv/h) where a species occurred. Species with missing information on percentage of yearlings could not be aged according to criteria compiled in ref. [37]. See Methods for sources. (DOC) [file pone.0016862.s001.doc]

Table S1. Species names, number of individuals captured, brain volume (mm^3^), brain mass (g), body mass (g), and age distribution (% yearlings) and range in radiation levels (μSv/h) where a species occurred. Species with missing information on percentage of yearlings could not be aged according to criteria compiled in ref. [37]. See Methods for sources.

| Species | No. individuals captured | Brain volume (mm^3^) | Brain mass (g) | Body mass (g) | Pct. yearlings | Range in radiation (μSv/h) |
| --- | --- | --- | --- | --- | --- | --- |
| *Acrocephalus arundinaceus* | 1 | 3787 | . | 30.35 |  | 0.82 |
| *Acrocephalus palustris* | 2 | 1611 | 0.52 | 12.00 |  | 0.85-0.91 |
| *Acrocephalus scirpaceus* | 1 | 1501 | 0.58 | 11.80 |  | 15.21 |
| *Aegitahlos caudatus* | 2 | 1156 | 0.42 | 8.80 |  | 94.61 |
| *Anthus trivialis* | 20 | 1928 | 0.62 | 23.40 |  | 6.10-92.90 |
| *Caprimulgus europaeus* | 1 | 5617 | 0.73 | 85.00 |  | 15.50 |
| *Carduelis chloris* | 1 | 1480 | 0.88 | 27.65 | 100 | 16.47 |
| *Carduelis spinus* | 2 | 1215 | 0.52 | 13.80 | 0 | 0.02 |
| *Certhia familiaris* | 5 | 1162 | 0.50 | 9.15 |  | 0.02-6.11 |
| *Coccothraustes coccothraustes* | 20 | 4743 | 1.69 | 54.70 |  | 0.02-84.00 |
| *Delichon urbica* | 2 | 1560 | 0.43 | 19.55 |  | 0.05 |
| *Dendrocopos leucotos* | 4 | 9504 | . | 107.00 |  | 15.94-74.06 |
| *Dendrocopos major* | 3 | 9260 | 2.58 | 89.65 |  | 0.02 |
| *Dendrocopos minor* | 4 | 3088 | 1.20 | 25.50 |  | 0.02-16.00 |
| *Emberiza citrinella* | 2 | 1851 | 0.77 | 26.75 | 50 | 0.86-8.50 |
| *Erithacus rubecula* | 33 | 2124 | 0.62 | 16.35 | 52 | 0.02-73.48 |
| *Ficedula parva* | 2 | 1309 | . | 11.70 | 0 | 0.02 |
| *Fringilla coelebs* | 57 | 1931 | 0.81 | 24.20 | 28 | 0.02-52.38 |
| *Garrulus glandarius* | 5 | 14614 | 3.97 | 161.70 | 75 | 0.02-46.16 |
| *Hippolais icterina* | 5 | 1700 | 0.48 | 13.30 |  | 7.56-25.90 |
| *Hirundo rustica* | 129 | 1953 | 0.55 | 19.10 | 59 | 0.02-2.90 |
| *Jynx torquilla* | 1 | 2551 | 0.85 | 37.35 | 36 | 23.56 |
| *Lanius collurio* | 25 | 3569 | 1.04 | 30.70 |  | 0.05-87.10 |
| *Locustella fluviatilis* | 1 | 1708 | . | 18.80 |  | 0.94 |
| *Lullula arborea* | 2 | 2091 | . | 30.05 |  | 6.85-8.94 |
| *Luscinia luscinia* | 14 | 2471 | 0.75 | 25.00 |  | 0.02-12.11 |
| *Motacilla alba* | 6 | 1841 | 0.53 | 20.75 | 67 | 0.18-21.19 |
| *Oriolus oriolus* | 1 | 5548 | 1.31 | 68.50 | 100 | 9.31 |
| *Parus caeruleus* | 3 | 1384 | 0.60 | 11.75 | 67 | 0.02-18.35 |
| *Parus cristatus* | 4 | 1557 | 0.66 | 11.15 |  | 0.02-10.23 |
| *Parus major* | 24 | 2323 | 0.85 | 18.50 | 58 | 0.02-85.90 |
| *Parus montanus* | 2 | 1875 | 0.79 | 11.65 |  | 12.34-25.57 |
| *Parus palustris* | 6 | 1478 | 0.72 | 11.90 | 100 | 0.02-25.00 |
| *Phoenicurus ochruros* | 7 | 1843 | 0.55 | 16.00 |  | 0.02-25.51 |
| *Phoenicurus phoenicurus* | 1 | 1942 | 0.49 | 15.90 | 0 | 4.02 |
| *Phylloscopus collybita* | 7 | 1225 | 0.36 | 7.70 |  | 0.02-84.00 |
| *Phylloscopus sibilatrix* | 15 | 1191 | . | 9.10 |  | 0.02-77.28 |
| *Phylloscopus trochilus* | 3 | 855 | 0.31 | 9.35 |  | 7.59-8.59 |
| *Sitta europaea* | 3 | 2630 | 0.98 | 23.90 |  | 0.02 |
| *Sylvia atricapilla* | 15 | 1798 | 0.63 | 18.85 |  | 0.02-12.51 |
| *Sylvia borin* | 6 | 2140 | 0.58 | 19.05 |  | 0.02-10.10 |
| *Sylvia communis* | 9 | 1775 | 0.52 | 14.50 |  | 0.66-18.86 |
| *Sylvia nisoria* | 13 | 2728 | . | 24.35 | 33 | 0.68-25.60 |
| *Troglodytes troglodytes* | 2 | 1361 | 0.47 | 8.90 | 0 | 0.02 |
| *Turdus merula* | 44 | 6497 | 1.94 | 95.85 | 61 | 0.02-92.32 |
| *Turdus philomelos* | 24 | 5294 | 1.59 | 70.50 | 83 | 0.02-72.01 |
| *Turdus viscivorus* | 4 | 7830 | 2.40 | 117.80 | 100 | 5.09-62.66 |
| *Upupa epops* | 3 | 4909 | 1.05 | 67.05 |  | 1.83-1.95 |
