# Supplementary material for: Chernobyl Birds Have Smaller Brains
Source: PLoS One. 2011 Feb 4;6(2):e16862. doi: 10.1371/journal.pone.0016862 (PMC3033907; doi:10.1371/journal.pone.0016862)
Supplement: Table S2 — Data on background radiation (μSv/h), body mass (g), beak length (mm) and head volume (mm3) for birds from the Chernobyl region. See Methods for further details. (DOC) [file pone.0016862.s002.doc]

Table S2. Data on background radiation (μSv/h), body mass (g), beak length (mm) and head volume (mm^3^) for birds from the Chernobyl region. See Methods for further details.

| Species | Radiation (μSv/h) | log Body mass (x 10, g) | log Beak length (x 100, mm) | log Head volume (mm^3^) |
| --- | --- | --- | --- | --- |
| *Acrocephalus palustris* | 0.91 | 2.376 | 3.073 | 3.401 |
| *Acrocephalus palustris* | 0.85 | 2.408 | 3.075 | 3.386 |
| *Anthus trivialis* | 11.86 | 2.349 | 3.069 | 3.438 |
| *Anthus trivialis* | 85.28 | 2.359 | 3.127 | 3.416 |
| *Anthus trivialis* | 21.60 | 2.359 | 3.088 | 3.348 |
| *Anthus trivialis* | 22.41 | 2.363 | 3.112 | 3.337 |
| *Anthus trivialis* | 7.62 | 2.372 | 3.037 | 3.379 |
| *Anthus trivialis* | 12.83 | 2.372 | 3.079 | 3.376 |
| *Anthus trivialis* | 13.21 | 2.374 | 3.097 | 3.456 |
| *Anthus trivialis* | 48.40 | 2.378 | 3.018 | 3.359 |
| *Anthus trivialis* | 8.25 | 2.384 | 3.058 | 3.390 |
| *Anthus trivialis* | 92.90 | 2.386 | 3.095 | 3.382 |
| *Anthus trivialis* | 27.80 | 2.390 | 3.046 | 3.417 |
| *Anthus trivialis* | 81.12 | 2.392 | 3.067 | 3.343 |
| *Anthus trivialis* | 19.19 | 2.392 | 3.050 | 3.382 |
| *Anthus trivialis* | 66.02 | 2.394 | 3.062 | 3.382 |
| *Anthus trivialis* | 25.45 | 2.394 | 3.131 | 3.468 |
| *Anthus trivialis* | 6.10 | 2.411 | 3.066 | 3.359 |
| *Anthus trivialis* | 7.10 | 2.420 | 3.092 | 3.318 |
| *Anthus trivialis* | 34.50 | 2.424 | 3.067 | 3.402 |
| *Anthus trivialis* | 27.42 | 2.452 | 3.083 | 3.451 |
| *Anthus trivialis* | 41.10 | 2.477 | 3.041 | 3.365 |
| *Certhia familiaris* | 5.70 | 2.358 | 3.021 | 3.458 |
| *Certhia familiaris* | 0.02 | 2.374 | 3.086 | 3.395 |
| *Certhia familiaris* | 0.02 | 2.395 | 3.071 | 3.371 |
| *Certhia familiaris* | 0.02 | 2.405 | 3.091 | 3.326 |
| *Certhia familiaris* | 6.11 | 2.429 | 3.102 | 3.416 |
| *Coccothraustes coccothraustes* | 31.31 | 2.338 | 3.088 | 3.380 |
| *Coccothraustes coccothraustes* | 0.02 | 2.352 | 3.032 | 3.409 |
| *Coccothraustes coccothraustes* | 7.94 | 2.357 | 3.095 | 3.380 |
| *Coccothraustes coccothraustes* | 0.02 | 2.357 | 3.055 | 3.424 |
| *Coccothraustes coccothraustes* | 0.02 | 2.358 | 3.036 | 3.414 |
| *Coccothraustes coccothraustes* | 21.40 | 2.359 | 3.031 | 3.370 |
| *Coccothraustes coccothraustes* | 0.02 | 2.380 | 3.121 | 3.365 |
| *Coccothraustes coccothraustes* | 0.02 | 2.387 | 3.048 | 3.348 |
| *Coccothraustes coccothraustes* | 21.40 | 2.391 | 3.076 | 3.402 |
| *Coccothraustes coccothraustes* | 0.78 | 2.391 | 3.044 | 3.433 |
| *Coccothraustes coccothraustes* | 6.17 | 2.392 | 3.111 | 3.360 |
| *Coccothraustes coccothraustes* | 7.51 | 2.392 | 3.068 | 3.377 |
| *Coccothraustes coccothraustes* | 0.02 | 2.396 | 3.036 | 3.397 |
| *Coccothraustes coccothraustes* | 0.97 | 2.405 | 3.093 | 3.416 |
| *Coccothraustes coccothraustes* | 0.02 | 2.406 | 3.128 | 3.369 |
| *Coccothraustes coccothraustes* | 15.13 | 2.419 | 3.107 | 3.409 |
| *Coccothraustes coccothraustes* | 0.02 | 2.420 | 3.077 | 3.389 |
| *Coccothraustes coccothraustes* | 0.02 | 2.434 | 3.105 | 3.424 |
| *Coccothraustes coccothraustes* | 84.00 | 2.437 | 3.080 | 3.411 |
| *Coccothraustes coccothraustes* | 0.02 | 2.470 | 3.054 | 3.390 |
| *Dendrocopos leucotos* | 17.66 | 2.363 | 3.049 | 3.320 |
| *Dendrocopos leucotos* | 15.94 | 2.392 | 3.115 | 3.419 |
| *Dendrocopos leucotos* | 74.06 | 2.392 | 3.043 | 3.449 |
| *Dendrocopos leucotos* | 74.06 | 2.421 | 3.091 | 3.385 |
| *Dendrocopos minor* | 0.84 | 2.368 | 3.129 | 3.379 |
| *Dendrocopos minor* | 0.89 | 2.378 | 3.105 | 3.394 |
| *Dendrocopos minor* | 16.00 | 2.411 | 3.053 | 3.420 |
| *Dendrocopos minor* | 0.02 | 2.411 | 3.010 | 3.381 |
| *Emberiza citrinella* | 8.50 | 2.352 | 3.068 | 3.388 |
| *Emberiza citrinella* | 0.86 | 2.432 | 3.080 | 3.399 |
| *Erithacus rubecula* | 0.02 | 2.351 | 3.087 | 3.338 |
| *Erithacus rubecula* | 73.48 | 2.354 | 3.081 | 3.435 |
| *Erithacus rubecula* | 3.78 | 2.357 | 3.070 | 3.383 |
| *Erithacus rubecula* | 1.11 | 2.357 | 3.056 | 3.442 |
| *Erithacus rubecula* | 14.76 | 2.359 | 3.088 | 3.295 |
| *Erithacus rubecula* | 62.82 | 2.362 | 3.051 | 3.315 |
| *Erithacus rubecula* | 1.73 | 2.362 | 3.094 | 3.414 |
| *Erithacus rubecula* | 0.02 | 2.362 | 3.074 | 3.384 |
| *Erithacus rubecula* | 11.94 | 2.365 | 3.059 | 3.385 |
| *Erithacus rubecula* | 22.88 | 2.365 | 3.136 | 3.462 |
| *Erithacus rubecula* | 72.82 | 2.368 | 3.088 | 3.438 |
| *Erithacus rubecula* | 0.02 | 2.368 | 3.097 | 3.376 |
| *Erithacus rubecula* | 58.11 | 2.374 | 3.094 | 3.320 |
| *Erithacus rubecula* | 0.02 | 2.376 | 3.091 | 3.346 |
| *Erithacus rubecula* | 8.32 | 2.379 | 3.109 | 3.358 |
| *Erithacus rubecula* | 3.22 | 2.379 | 3.100 | 3.409 |
| *Erithacus rubecula* | 8.10 | 2.379 | 3.087 | 3.362 |
| *Erithacus rubecula* | 4.19 | 2.379 | 3.076 | 3.315 |
| *Erithacus rubecula* | 7.08 | 2.379 | 3.098 | 3.379 |
| *Erithacus rubecula* | 6.80 | 2.382 | 3.006 | 3.410 |
| *Erithacus rubecula* | 0.02 | 2.382 | 3.048 | 3.418 |
| *Erithacus rubecula* | 0.02 | 2.390 | 3.076 | 3.429 |
| *Erithacus rubecula* | 2.03 | 2.406 | 3.082 | 3.423 |
| *Erithacus rubecula* | 0.02 | 2.406 | 3.043 | 3.463 |
| *Erithacus rubecula* | 8.00 | 2.411 | 3.073 | 3.404 |
| *Erithacus rubecula* | 2.90 | 2.411 | 3.055 | 3.400 |
| *Erithacus rubecula* | 23.91 | 2.419 | 3.104 | 3.387 |
| *Erithacus rubecula* | 8.76 | 2.431 | 3.055 | 3.345 |
| *Erithacus rubecula* | 10.70 | 2.431 | 3.075 | 3.430 |
| *Erithacus rubecula* | 55.04 | 2.455 | 3.048 | 3.399 |
| *Erithacus rubecula* | 0.02 | 2.459 | 3.072 | 3.428 |
| *Erithacus rubecula* | 0.02 | 2.459 | 3.053 | 3.438 |
| *Erithacus rubecula* | 13.41 | 2.519 | 3.023 | 3.393 |
| *Fringilla coelebs* | 13.12 | 2.322 | 3.085 | 3.312 |
| *Fringilla coelebs* | 0.02 | 2.340 | 3.087 | 3.313 |
| *Fringilla coelebs* | 2.92 | 2.345 | 2.998 | 3.376 |
| *Fringilla coelebs* | 17.27 | 2.358 | 3.092 | 3.384 |
| *Fringilla coelebs* | 2.03 | 2.358 | 3.073 | 3.357 |
| *Fringilla coelebs* | 0.02 | 2.358 | 3.031 | 3.435 |
| *Fringilla coelebs* | 0.02 | 2.363 | 3.002 | 3.371 |
| *Fringilla coelebs* | 13.26 | 2.365 | 3.087 | 3.389 |
| *Fringilla coelebs* | 0.02 | 2.365 | 3.081 | 3.433 |
| *Fringilla coelebs* | 7.59 | 2.367 | 3.041 | 3.295 |
| *Fringilla coelebs* | 0.02 | 2.367 | 3.067 | 3.406 |
| *Fringilla coelebs* | 45.05 | 2.369 | 3.087 | 3.377 |
| *Fringilla coelebs* | 26.00 | 2.371 | 3.069 | 3.378 |
| *Fringilla coelebs* | 18.36 | 2.371 | 3.066 | 3.378 |
| *Fringilla coelebs* | 0.02 | 2.371 | 3.029 | 3.431 |
| *Fringilla coelebs* | 8.90 | 2.376 | 3.059 | 3.447 |
| *Fringilla coelebs* | 5.16 | 2.376 | 3.079 | 3.379 |
| *Fringilla coelebs* | 52.38 | 2.380 | 3.095 | 3.383 |
| *Fringilla coelebs* | 25.50 | 2.380 | 3.083 | 3.353 |
| *Fringilla coelebs* | 0.02 | 2.380 | 3.068 | 3.419 |
| *Fringilla coelebs* | 0.78 | 2.380 | 3.086 | 3.371 |
| *Fringilla coelebs* | 9.03 | 2.382 | 3.091 | 3.415 |
| *Fringilla coelebs* | 27.00 | 2.384 | 3.064 | 3.429 |
| *Fringilla coelebs* | 17.16 | 2.384 | 3.083 | 3.438 |
| *Fringilla coelebs* | 0.02 | 2.384 | 3.070 | 3.436 |
| *Fringilla coelebs* | 4.04 | 2.386 | 3.057 | 3.338 |
| *Fringilla coelebs* | 11.31 | 2.388 | 3.086 | 3.424 |
| *Fringilla coelebs* | 0.02 | 2.388 | 3.088 | 3.390 |
| *Fringilla coelebs* | 0.02 | 2.388 | 3.080 | 3.397 |
| *Fringilla coelebs* | 0.02 | 2.388 | 3.094 | 3.388 |
| *Fringilla coelebs* | 30.19 | 2.390 | 3.085 | 3.379 |
| *Fringilla coelebs* | 45.05 | 2.390 | 3.085 | 3.429 |
| *Fringilla coelebs* | 20.00 | 2.394 | 3.098 | 3.389 |
| *Fringilla coelebs* | 0.02 | 2.394 | 3.041 | 3.472 |
| *Fringilla coelebs* | 0.02 | 2.400 | 3.093 | 3.360 |
| *Fringilla coelebs* | 0.02 | 2.400 | 3.072 | 3.317 |
| *Fringilla coelebs* | 0.02 | 2.400 | 3.090 | 3.443 |
| *Fringilla coelebs* | 21.56 | 2.404 | 3.070 | 3.425 |
| *Fringilla coelebs* | 0.02 | 2.406 | 3.107 | 3.399 |
| *Fringilla coelebs* | 0.02 | 2.408 | 3.058 | 3.367 |
| *Fringilla coelebs* | 0.02 | 2.408 | 3.115 | 3.372 |
| *Fringilla coelebs* | 0.02 | 2.408 | 3.044 | 3.409 |
| *Fringilla coelebs* | 17.27 | 2.410 | 3.090 | 3.460 |
| *Fringilla coelebs* | 11.12 | 2.410 | 3.109 | 3.416 |
| *Fringilla coelebs* | 5.65 | 2.410 | 3.066 | 3.411 |
| *Fringilla coelebs* | 0.02 | 2.410 | 3.084 | 3.377 |
| *Fringilla coelebs* | 0.02 | 2.410 | 3.072 | 3.420 |
| *Fringilla coelebs* | 5.33 | 2.416 | 3.080 | 3.402 |
| *Fringilla coelebs* | 5.65 | 2.418 | 3.091 | 3.366 |
| *Fringilla coelebs* | 1.61 | 2.422 | 3.027 | 3.397 |
| *Fringilla coelebs* | 7.35 | 2.425 | 3.081 | 3.440 |
| *Fringilla coelebs* | 4.60 | 2.427 | 3.082 | 3.390 |
| *Fringilla coelebs* | 0.02 | 2.427 | 3.058 | 3.361 |
| *Fringilla coelebs* | 0.02 | 2.429 | 3.098 | 3.398 |
| *Fringilla coelebs* | 12.39 | 2.444 | 3.075 | 3.354 |
| *Fringilla coelebs* | 0.74 | 2.461 | 3.094 | 3.397 |
| *Fringilla coelebs* | 2.21 | 2.488 | 3.088 | 3.384 |
| *Garrulus glandarius* | 14.30 | 2.392 | 3.051 | 3.357 |
| *Garrulus glandarius* | 46.16 | 2.392 | 3.099 | 3.429 |
| *Garrulus glandarius* | 18.80 | 2.392 | 3.104 | 3.376 |
| *Garrulus glandarius* | 13.39 | 2.392 | 3.072 | 3.372 |
| *Garrulus glandarius* | 0.02 | 2.392 | 3.046 | 3.432 |
| *Hippolais icterina* | 7.56 | 2.369 | 3.096 | 3.419 |
| *Hippolais icterina* | 10.33 | 2.369 | 3.069 | 3.358 |
| *Hippolais icterina* | 16.11 | 2.397 | 3.054 | 3.353 |
| *Hippolais icterina* | 13.39 | 2.400 | 3.056 | 3.434 |
| *Hippolais icterina* | 25.90 | 2.426 | 3.096 | 3.401 |
| *Hirundo rustica* | 0.05 | 2.320 | 3.054 | 3.333 |
| *Hirundo rustica* | 0.05 | 2.323 | 3.038 | 3.381 |
| *Hirundo rustica* | 0.02 | 2.323 | 3.050 | 3.316 |
| *Hirundo rustica* | 0.02 | 2.328 | 3.075 | 3.340 |
| *Hirundo rustica* | 0.02 | 2.331 | 3.108 | 3.363 |
| *Hirundo rustica* | 0.05 | 2.339 | 3.029 | 3.350 |
| *Hirundo rustica* | 0.05 | 2.339 | 3.017 | 3.343 |
| *Hirundo rustica* | 0.02 | 2.339 | 3.084 | 3.406 |
| *Hirundo rustica* | 0.02 | 2.344 | 3.085 | 3.322 |
| *Hirundo rustica* | 2.90 | 2.346 | 2.992 | 3.453 |
| *Hirundo rustica* | 2.90 | 2.346 | 3.116 | 3.398 |
| *Hirundo rustica* | 0.05 | 2.346 | 3.105 | 3.394 |
| *Hirundo rustica* | 2.90 | 2.349 | 3.109 | 3.403 |
| *Hirundo rustica* | 0.05 | 2.349 | 3.091 | 3.356 |
| *Hirundo rustica* | 0.05 | 2.351 | 3.119 | 3.404 |
| *Hirundo rustica* | 0.02 | 2.351 | 3.055 | 3.378 |
| *Hirundo rustica* | 0.02 | 2.351 | 3.078 | 3.425 |
| *Hirundo rustica* | 2.90 | 2.354 | 3.025 | 3.336 |
| *Hirundo rustica* | 0.05 | 2.359 | 3.038 | 3.387 |
| *Hirundo rustica* | 0.05 | 2.364 | 3.073 | 3.397 |
| *Hirundo rustica* | 0.05 | 2.364 | 3.061 | 3.400 |
| *Hirundo rustica* | 0.05 | 2.364 | 3.043 | 3.391 |
| *Hirundo rustica* | 2.90 | 2.364 | 3.039 | 3.403 |
| *Hirundo rustica* | 0.05 | 2.364 | 3.113 | 3.402 |
| *Hirundo rustica* | 0.05 | 2.364 | 3.058 | 3.356 |
| *Hirundo rustica* | 0.02 | 2.364 | 3.068 | 3.376 |
| *Hirundo rustica* | 0.02 | 2.364 | 3.060 | 3.396 |
| *Hirundo rustica* | 2.90 | 2.366 | 3.050 | 3.325 |
| *Hirundo rustica* | 0.02 | 2.366 | 3.102 | 3.414 |
| *Hirundo rustica* | 0.02 | 2.369 | 3.107 | 3.439 |
| *Hirundo rustica* | 0.02 | 2.369 | 3.078 | 3.334 |
| *Hirundo rustica* | 0.02 | 2.369 | 3.089 | 3.395 |
| *Hirundo rustica* | 0.02 | 2.369 | 3.076 | 3.392 |
| *Hirundo rustica* | 0.02 | 2.369 | 3.099 | 3.323 |
| *Hirundo rustica* | 0.02 | 2.371 | 3.031 | 3.363 |
| *Hirundo rustica* | 0.02 | 2.371 | 3.025 | 3.358 |
| *Hirundo rustica* | 0.02 | 2.371 | 3.057 | 3.415 |
| *Hirundo rustica* | 0.02 | 2.371 | 3.114 | 3.459 |
| *Hirundo rustica* | 2.90 | 2.373 | 3.046 | 3.436 |
| *Hirundo rustica* | 0.05 | 2.373 | 3.068 | 3.399 |
| *Hirundo rustica* | 0.05 | 2.373 | 3.044 | 3.435 |
| *Hirundo rustica* | 0.02 | 2.373 | 3.103 | 3.403 |
| *Hirundo rustica* | 0.02 | 2.373 | 3.037 | 3.405 |
| *Hirundo rustica* | 0.02 | 2.373 | 3.097 | 3.414 |
| *Hirundo rustica* | 2.90 | 2.376 | 3.019 | 3.409 |
| *Hirundo rustica* | 0.05 | 2.376 | 3.108 | 3.407 |
| *Hirundo rustica* | 0.02 | 2.376 | 3.082 | 3.403 |
| *Hirundo rustica* | 0.02 | 2.376 | 3.137 | 3.410 |
| *Hirundo rustica* | 0.05 | 2.378 | 3.033 | 3.333 |
| *Hirundo rustica* | 2.90 | 2.378 | 3.075 | 3.398 |
| *Hirundo rustica* | 0.05 | 2.381 | 3.082 | 3.443 |
| *Hirundo rustica* | 0.02 | 2.381 | 3.135 | 3.426 |
| *Hirundo rustica* | 2.90 | 2.383 | 3.090 | 3.401 |
| *Hirundo rustica* | 2.90 | 2.385 | 3.050 | 3.362 |
| *Hirundo rustica* | 0.02 | 2.385 | 3.098 | 3.428 |
| *Hirundo rustica* | 0.05 | 2.388 | 3.093 | 3.368 |
| *Hirundo rustica* | 0.05 | 2.388 | 3.093 | 3.364 |
| *Hirundo rustica* | 0.05 | 2.388 | 3.100 | 3.374 |
| *Hirundo rustica* | 0.05 | 2.388 | 3.080 | 3.451 |
| *Hirundo rustica* | 0.05 | 2.390 | 3.026 | 3.367 |
| *Hirundo rustica* | 0.02 | 2.390 | 3.096 | 3.363 |
| *Hirundo rustica* | 0.02 | 2.390 | 3.094 | 3.344 |
| *Hirundo rustica* | 0.02 | 2.390 | 3.113 | 3.426 |
| *Hirundo rustica* | 0.02 | 2.390 | 3.045 | 3.509 |
| *Hirundo rustica* | 0.02 | 2.392 | 3.089 | 3.449 |
| *Hirundo rustica* | 0.02 | 2.392 | 3.084 | 3.341 |
| *Hirundo rustica* | 0.02 | 2.392 | 3.105 | 3.355 |
| *Hirundo rustica* | 0.02 | 2.392 | 3.049 | 3.441 |
| *Hirundo rustica* | 0.02 | 2.392 | 3.074 | 3.370 |
| *Hirundo rustica* | 0.05 | 2.394 | 3.083 | 3.409 |
| *Hirundo rustica* | 0.05 | 2.394 | 3.039 | 3.415 |
| *Hirundo rustica* | 0.02 | 2.394 | 3.085 | 3.441 |
| *Hirundo rustica* | 0.02 | 2.397 | 3.099 | 3.426 |
| *Hirundo rustica* | 0.02 | 2.397 | 3.112 | 3.406 |
| *Hirundo rustica* | 0.02 | 2.397 | 3.089 | 3.417 |
| *Hirundo rustica* | 2.90 | 2.399 | 3.079 | 3.402 |
| *Hirundo rustica* | 0.05 | 2.399 | 3.064 | 3.450 |
| *Hirundo rustica* | 0.02 | 2.399 | 3.066 | 3.365 |
| *Hirundo rustica* | 0.02 | 2.399 | 3.070 | 3.442 |
| *Hirundo rustica* | 0.05 | 2.401 | 3.106 | 3.364 |
| *Hirundo rustica* | 0.05 | 2.403 | 3.063 | 3.370 |
| *Hirundo rustica* | 0.05 | 2.403 | 3.051 | 3.371 |
| *Hirundo rustica* | 0.05 | 2.403 | 3.046 | 3.412 |
| *Hirundo rustica* | 0.02 | 2.403 | 3.023 | 3.392 |
| *Hirundo rustica* | 0.02 | 2.403 | 3.033 | 3.416 |
| *Hirundo rustica* | 2.90 | 2.408 | 3.039 | 3.478 |
| *Hirundo rustica* | 0.05 | 2.410 | 3.006 | 3.450 |
| *Hirundo rustica* | 0.05 | 2.410 | 3.054 | 3.398 |
| *Hirundo rustica* | 0.02 | 2.410 | 3.077 | 3.419 |
| *Hirundo rustica* | 0.02 | 2.410 | 3.074 | 3.401 |
| *Hirundo rustica* | 0.02 | 2.412 | 3.091 | 3.401 |
| *Hirundo rustica* | 0.05 | 2.414 | 3.083 | 3.351 |
| *Hirundo rustica* | 0.05 | 2.414 | 3.113 | 3.401 |
| *Hirundo rustica* | 2.90 | 2.417 | 3.059 | 3.374 |
| *Hirundo rustica* | 0.02 | 2.419 | 3.099 | 3.427 |
| *Hirundo rustica* | 0.02 | 2.419 | 3.075 | 3.358 |
| *Hirundo rustica* | 0.05 | 2.421 | 3.090 | 3.389 |
| *Hirundo rustica* | 0.05 | 2.425 | 3.045 | 3.343 |
| *Hirundo rustica* | 0.02 | 2.425 | 3.097 | 3.507 |
| *Hirundo rustica* | 0.05 | 2.427 | 3.075 | 3.364 |
| *Hirundo rustica* | 0.02 | 2.427 | 3.104 | 3.326 |
| *Hirundo rustica* | 0.02 | 2.427 | 3.107 | 3.463 |
| *Hirundo rustica* | 0.05 | 2.431 | 3.088 | 3.440 |
| *Hirundo rustica* | 0.05 | 2.431 | 3.040 | 3.432 |
| *Hirundo rustica* | 0.05 | 2.431 | 3.080 | 3.411 |
| *Hirundo rustica* | 0.05 | 2.431 | 3.085 | 3.428 |
| *Hirundo rustica* | 0.02 | 2.431 | 3.106 | 3.400 |
| *Hirundo rustica* | 0.02 | 2.431 | 3.073 | 3.458 |
| *Hirundo rustica* | 0.05 | 2.434 | 3.069 | 3.435 |
| *Hirundo rustica* | 0.05 | 2.436 | 3.106 | 3.412 |
| *Hirundo rustica* | 0.02 | 2.438 | 3.102 | 3.400 |
| *Hirundo rustica* | 0.05 | 2.442 | 3.101 | 3.341 |
| *Hirundo rustica* | 0.05 | 2.442 | 3.069 | 3.392 |
| *Hirundo rustica* | 0.05 | 2.446 | 3.053 | 3.405 |
| *Hirundo rustica* | 0.05 | 2.446 | 3.110 | 3.441 |
| *Hirundo rustica* | 0.05 | 2.452 | 3.113 | 3.364 |
| *Hirundo rustica* | 0.05 | 2.458 | 3.049 | 3.362 |
| *Hirundo rustica* | 0.05 | 2.462 | 3.076 | 3.374 |
| *Hirundo rustica* | 0.05 | 2.466 | 3.071 | 3.428 |
| *Hirundo rustica* | 0.05 | 2.467 | 3.100 | 3.435 |
| *Hirundo rustica* | 0.05 | 2.471 | 3.080 | 3.406 |
| *Hirundo rustica* | 0.05 | 2.475 | 3.070 | 3.401 |
| *Hirundo rustica* | 0.05 | 2.479 | 3.044 | 3.356 |
| *Lanius collurio* | 0.05 | 2.315 | 3.030 | 3.336 |
| *Lanius collurio* | 28.89 | 2.324 | 3.066 | 3.397 |
| *Lanius collurio* | 82.50 | 2.339 | 3.184 | 3.351 |
| *Lanius collurio* | 12.86 | 2.347 | 3.091 | 3.393 |
| *Lanius collurio* | 0.65 | 2.347 | 3.045 | 3.311 |
| *Lanius collurio* | 12.56 | 2.350 | 3.070 | 3.328 |
| *Lanius collurio* | 7.94 | 2.352 | 3.050 | 3.463 |
| *Lanius collurio* | 8.36 | 2.352 | 3.101 | 3.385 |
| *Lanius collurio* | 1.15 | 2.353 | 3.053 | 3.415 |
| *Lanius collurio* | 0.95 | 2.353 | 3.053 | 3.426 |
| *Lanius collurio* | 12.06 | 2.355 | 3.105 | 3.416 |
| *Lanius collurio* | 15.83 | 2.358 | 3.062 | 3.395 |
| *Lanius collurio* | 0.83 | 2.360 | 3.081 | 3.357 |
| *Lanius collurio* | 84.00 | 2.369 | 3.072 | 3.355 |
| *Lanius collurio* | 14.51 | 2.371 | 3.025 | 3.397 |
| *Lanius collurio* | 20.00 | 2.421 | 3.044 | 3.436 |
| *Lanius collurio* | 87.10 | 2.424 | 3.044 | 3.399 |
| *Lanius collurio* | 0.58 | 2.448 | 3.089 | 3.389 |
| *Lanius collurio* | 10.91 | 2.450 | 3.062 | 3.410 |
| *Lanius collurio* | 9.75 | 2.457 | 3.075 | 3.433 |
| *Lanius collurio* | 15.83 | 2.458 | 3.076 | 3.338 |
| *Lanius collurio* | 77.34 | 2.465 | 3.177 | 3.396 |
| *Lanius collurio* | 0.99 | 2.471 | 3.060 | 3.432 |
| *Lanius collurio* | 13.98 | 2.479 | 3.057 | 3.448 |
| *Lanius collurio* | 0.86 | 2.485 | 3.084 | 3.380 |
| *Lullula arborea* | 6.85 | 2.344 | 3.051 | 3.389 |
| *Lullula arborea* | 8.94 | 2.440 | 3.097 | 3.398 |
| *Luscinia luscinia* | 0.88 | 2.310 | 3.050 | 3.395 |
| *Luscinia luscinia* | 1.26 | 2.323 | 3.074 | 3.387 |
| *Luscinia luscinia* | 0.96 | 2.357 | 3.045 | 3.352 |
| *Luscinia luscinia* | 1.33 | 2.371 | 3.081 | 3.461 |
| *Luscinia luscinia* | 1.21 | 2.375 | 3.099 | 3.356 |
| *Luscinia luscinia* | 0.91 | 2.376 | 3.073 | 3.392 |
| *Luscinia luscinia* | 2.66 | 2.378 | 3.058 | 3.374 |
| *Luscinia luscinia* | 10.50 | 2.386 | 3.076 | 3.414 |
| *Luscinia luscinia* | 11.08 | 2.400 | 3.098 | 3.365 |
| *Luscinia luscinia* | 0.02 | 2.409 | 3.055 | 3.433 |
| *Luscinia luscinia* | 1.19 | 2.409 | 3.085 | 3.405 |
| *Luscinia luscinia* | 12.11 | 2.426 | 3.070 | 3.393 |
| *Luscinia luscinia* | 9.37 | 2.442 | 3.070 | 3.394 |
| *Luscinia luscinia* | 10.40 | 2.527 | 3.106 | 3.387 |
| *Motacilla alba* | 3.65 | 2.351 | 3.057 | 3.359 |
| *Motacilla alba* | 21.19 | 2.364 | 3.063 | 3.261 |
| *Motacilla alba* | 0.18 | 2.402 | 3.066 | 3.447 |
| *Motacilla alba* | 4.60 | 2.408 | 3.051 | 3.450 |
| *Motacilla alba* | 21.12 | 2.410 | 3.102 | 3.408 |
| *Motacilla alba* | 3.69 | 2.418 | 3.106 | 3.434 |
| *Parus caeruleus* | 0.02 | 2.368 | 3.090 | 3.390 |
| *Parus caeruleus* | 18.35 | 2.381 | 3.104 | 3.413 |
| *Parus caeruleus* | 0.02 | 2.426 | 3.030 | 3.377 |
| *Parus cristatus* | 0.02 | 2.370 | 3.112 | 3.370 |
| *Parus cristatus* | 8.61 | 2.395 | 3.052 | 3.362 |
| *Parus cristatus* | 10.23 | 2.399 | 3.051 | 3.388 |
| *Parus cristatus* | 6.92 | 2.403 | 3.081 | 3.453 |
| *Parus major* | 0.02 | 2.300 | 3.100 | 3.311 |
| *Parus major* | 17.50 | 2.354 | 3.033 | 3.342 |
| *Parus major* | 14.19 | 2.354 | 3.088 | 3.335 |
| *Parus major* | 5.54 | 2.362 | 3.052 | 3.385 |
| *Parus major* | 16.41 | 2.362 | 3.062 | 3.387 |
| *Parus major* | 14.19 | 2.362 | 3.107 | 3.358 |
| *Parus major* | 5.43 | 2.370 | 3.048 | 3.393 |
| *Parus major* | 29.75 | 2.378 | 3.068 | 3.365 |
| *Parus major* | 2.45 | 2.380 | 3.090 | 3.389 |
| *Parus major* | 5.49 | 2.383 | 3.121 | 3.323 |
| *Parus major* | 0.02 | 2.388 | 3.051 | 3.384 |
| *Parus major* | 0.02 | 2.388 | 3.063 | 3.374 |
| *Parus major* | 7.63 | 2.395 | 3.095 | 3.387 |
| *Parus major* | 0.02 | 2.395 | 3.077 | 3.406 |
| *Parus major* | 0.02 | 2.395 | 3.034 | 3.418 |
| *Parus major* | 4.18 | 2.402 | 3.082 | 3.373 |
| *Parus major* | 9.21 | 2.407 | 3.055 | 3.536 |
| *Parus major* | 0.02 | 2.414 | 3.045 | 3.407 |
| *Parus major* | 0.02 | 2.417 | 3.054 | 3.395 |
| *Parus major* | 1.32 | 2.426 | 3.104 | 3.443 |
| *Parus major* | 1.39 | 2.439 | 3.112 | 3.545 |
| *Parus major* | 0.02 | 2.441 | 3.045 | 3.433 |
| *Parus major* | 85.90 | 2.446 | 3.075 | 3.341 |
| *Parus major* | 10.55 | 2.448 | 3.119 | 3.410 |
| *Parus montanus* | 12.34 | 2.388 | 3.090 | 3.389 |
| *Parus montanus* | 27.57 | 2.396 | 3.058 | 3.398 |
| *Parus palustris* | 0.02 | 2.350 | 2.999 | 3.385 |
| *Parus palustris* | 7.57 | 2.359 | 3.054 | 3.276 |
| *Parus palustris* | 0.02 | 2.377 | 3.028 | 3.403 |
| *Parus palustris* | 8.56 | 2.389 | 3.132 | 3.337 |
| *Parus palustris* | 0.02 | 2.421 | 3.076 | 3.463 |
| *Parus palustris* | 25.00 | 2.455 | 3.156 | 3.497 |
| *Phoenicurus ochruros* | 20.13 | 2.346 | 3.033 | 3.403 |
| *Phoenicurus ochruros* | 25.51 | 2.387 | 3.008 | 3.432 |
| *Phoenicurus ochruros* | 0.02 | 2.387 | 3.051 | 3.460 |
| *Phoenicurus ochruros* | 7.61 | 2.393 | 3.071 | 3.409 |
| *Phoenicurus ochruros* | 0.05 | 2.399 | 3.035 | 3.445 |
| *Phoenicurus ochruros* | 0.05 | 2.408 | 3.301 | 3.334 |
| *Phoenicurus ochruros* | 25.50 | 2.424 | 3.020 | 3.371 |
| *Phylloscopus collybita* | 0.02 | 2.364 | 3.073 | 3.386 |
| *Phylloscopus collybita* | 1.00 | 2.370 | 3.066 | 3.322 |
| *Phylloscopus collybita* | 0.88 | 2.370 | 3.029 | 3.351 |
| *Phylloscopus collybita* | 0.02 | 2.376 | 3.079 | 3.322 |
| *Phylloscopus collybita* | 84.00 | 2.405 | 3.082 | 3.523 |
| *Phylloscopus collybita* | 0.02 | 2.411 | 3.075 | 3.321 |
| *Phylloscopus collybita* | 0.02 | 2.448 | 3.116 | 3.388 |
| *Phylloscopus sibilatrix* | 0.02 | 2.337 | 3.046 | 3.303 |
| *Phylloscopus sibilatrix* | 77.28 | 2.363 | 3.064 | 3.266 |
| *Phylloscopus sibilatrix* | 17.50 | 2.368 | 3.076 | 3.319 |
| *Phylloscopus sibilatrix* | 68.01 | 2.372 | 3.083 | 3.356 |
| *Phylloscopus sibilatrix* | 0.02 | 2.372 | 3.056 | 3.330 |
| *Phylloscopus sibilatrix* | 0.02 | 2.377 | 3.092 | 3.374 |
| *Phylloscopus sibilatrix* | 4.65 | 2.382 | 3.058 | 3.400 |
| *Phylloscopus sibilatrix* | 57.08 | 2.382 | 3.112 | 3.386 |
| *Phylloscopus sibilatrix* | 0.02 | 2.382 | 3.115 | 3.399 |
| *Phylloscopus sibilatrix* | 54.19 | 2.396 | 3.097 | 3.367 |
| *Phylloscopus sibilatrix* | 0.02 | 2.396 | 3.057 | 3.455 |
| *Phylloscopus sibilatrix* | 0.02 | 2.418 | 3.071 | 3.346 |
| *Phylloscopus sibilatrix* | 0.02 | 2.427 | 3.042 | 3.519 |
| *Phylloscopus sibilatrix* | 0.02 | 2.439 | 3.101 | 3.439 |
| *Phylloscopus sibilatrix* | 45.80 | 2.467 | 3.043 | 3.433 |
| *Phylloscopus trochilus* | 8.20 | 2.373 | 3.025 | 3.292 |
| *Phylloscopus trochilus* | 8.59 | 2.394 | 3.104 | 3.457 |
| *Phylloscopus trochilus* | 7.59 | 2.409 | 3.094 | 3.431 |
| *Sylvia atricapilla* | 0.02 | 2.317 | 3.093 | 3.388 |
| *Sylvia atricapilla* | 0.02 | 2.320 | 3.138 | 3.380 |
| *Sylvia atricapilla* | 10.35 | 2.351 | 3.051 | 3.318 |
| *Sylvia atricapilla* | 0.02 | 2.351 | 3.109 | 3.430 |
| *Sylvia atricapilla* | 0.02 | 2.354 | 3.132 | 3.410 |
| *Sylvia atricapilla* | 0.02 | 2.377 | 3.002 | 3.416 |
| *Sylvia atricapilla* | 12.51 | 2.385 | 3.048 | 3.447 |
| *Sylvia atricapilla* | 0.02 | 2.385 | 3.081 | 3.388 |
| *Sylvia atricapilla* | 0.02 | 2.387 | 3.077 | 3.436 |
| *Sylvia atricapilla* | 0.02 | 2.397 | 3.038 | 3.366 |
| *Sylvia atricapilla* | 8.24 | 2.428 | 3.077 | 3.420 |
| *Sylvia atricapilla* | 4.05 | 2.446 | 3.078 | 3.349 |
| *Sylvia atricapilla* | 0.02 | 2.446 | 3.039 | 3.378 |
| *Sylvia atricapilla* | 12.05 | 2.450 | 3.091 | 3.372 |
| *Sylvia atricapilla* | 0.02 | 2.485 | 3.059 | 3.403 |
| *Sylvia borin* | 0.75 | 2.337 | 3.062 | 3.392 |
| *Sylvia borin* | 0.60 | 2.382 | 3.103 | 3.381 |
| *Sylvia borin* | 0.02 | 2.390 | 3.105 | 3.377 |
| *Sylvia borin* | 10.10 | 2.395 | 3.019 | 3.378 |
| *Sylvia borin* | 8.42 | 2.404 | 3.051 | 3.456 |
| *Sylvia borin* | 0.02 | 2.443 | 3.106 | 3.376 |
| *Sylvia communis* | 11.15 | 2.364 | 3.036 | 3.410 |
| *Sylvia communis* | 11.15 | 2.370 | 3.040 | 3.426 |
| *Sylvia communis* | 18.41 | 2.376 | 3.063 | 3.401 |
| *Sylvia communis* | 18.86 | 2.379 | 3.107 | 3.305 |
| *Sylvia communis* | 10.03 | 2.385 | 3.063 | 3.359 |
| *Sylvia communis* | 11.06 | 2.388 | 3.060 | 3.428 |
| *Sylvia communis* | 10.42 | 2.391 | 3.122 | 3.419 |
| *Sylvia communis* | 11.59 | 2.408 | 3.070 | 3.389 |
| *Sylvia communis* | 0.66 | 2.465 | 3.107 | 3.405 |
| *Sylvia nisoria* | 11.00 | 2.357 | 3.060 | 3.362 |
| *Sylvia nisoria* | 0.72 | 2.357 | 3.093 | 3.428 |
| *Sylvia nisoria* | 25.60 | 2.372 | 3.063 | 3.349 |
| *Sylvia nisoria* | 0.68 | 2.372 | 3.060 | 3.417 |
| *Sylvia nisoria* | 9.91 | 2.377 | 3.063 | 3.401 |
| *Sylvia nisoria* | 0.98 | 2.379 | 3.077 | 3.408 |
| *Sylvia nisoria* | 1.05 | 2.392 | 3.061 | 3.417 |
| *Sylvia nisoria* | 8.95 | 2.396 | 3.102 | 3.388 |
| *Sylvia nisoria* | 9.78 | 2.396 | 3.063 | 3.386 |
| *Sylvia nisoria* | 17.44 | 2.399 | 3.087 | 3.344 |
| *Sylvia nisoria* | 5.40 | 2.409 | 3.072 | 3.323 |
| *Sylvia nisoria* | 15.16 | 2.440 | 3.109 | 3.421 |
| *Sylvia nisoria* | 6.85 | 2.451 | 3.057 | 3.368 |
| *Turdus merula* | 0.05 | 2.372 | 3.041 | 3.390 |
| *Turdus merula* | 5.90 | 2.372 | 3.039 | 3.462 |
| *Turdus merula* | 8.22 | 2.377 | 3.043 | 3.391 |
| *Turdus merula* | 3.85 | 2.377 | 3.038 | 3.392 |
| *Turdus merula* | 7.55 | 2.381 | 3.042 | 3.343 |
| *Turdus merula* | 8.88 | 2.386 | 3.061 | 3.354 |
| *Turdus merula* | 7.03 | 2.390 | 3.037 | 3.363 |
| *Turdus merula* | 4.20 | 2.394 | 3.057 | 3.404 |
| *Turdus merula* | 3.58 | 2.394 | 3.105 | 3.379 |
| *Turdus merula* | 4.03 | 2.394 | 3.031 | 3.344 |
| *Turdus merula* | 12.45 | 2.394 | 3.062 | 3.403 |
| *Turdus merula* | 55.81 | 2.394 | 3.065 | 3.235 |
| *Turdus merula* | 84.90 | 2.394 | 3.055 | 3.424 |
| *Turdus merula* | 14.03 | 2.394 | 3.031 | 3.428 |
| *Turdus merula* | 64.04 | 2.394 | 3.075 | 3.383 |
| *Turdus merula* | 82.05 | 2.394 | 3.069 | 3.444 |
| *Turdus merula* | 86.55 | 2.394 | 3.077 | 3.376 |
| *Turdus merula* | 92.32 | 2.394 | 3.093 | 3.428 |
| *Turdus merula* | 20.25 | 2.394 | 3.101 | 3.400 |
| *Turdus merula* | 24.19 | 2.394 | 3.099 | 3.333 |
| *Turdus merula* | 73.02 | 2.394 | 3.081 | 3.424 |
| *Turdus merula* | 56.60 | 2.394 | 3.076 | 3.418 |
| *Turdus merula* | 14.56 | 2.394 | 3.100 | 3.359 |
| *Turdus merula* | 26.50 | 2.394 | 3.072 | 3.397 |
| *Turdus merula* | 4.31 | 2.394 | 3.078 | 3.376 |
| *Turdus merula* | 14.27 | 2.394 | 3.089 | 3.418 |
| *Turdus merula* | 10.97 | 2.394 | 3.084 | 3.403 |
| *Turdus merula* | 10.05 | 2.394 | 3.066 | 3.392 |
| *Turdus merula* | 16.43 | 2.394 | 3.065 | 3.354 |
| *Turdus merula* | 17.94 | 2.394 | 3.070 | 3.426 |
| *Turdus merula* | 10.54 | 2.394 | 3.062 | 3.469 |
| *Turdus merula* | 14.15 | 2.394 | 3.043 | 3.392 |
| *Turdus merula* | 0.02 | 2.394 | 3.055 | 3.323 |
| *Turdus merula* | 0.02 | 2.394 | 3.066 | 3.405 |
| *Turdus merula* | 0.02 | 2.394 | 3.028 | 3.421 |
| *Turdus merula* | 0.02 | 2.394 | 3.178 | 3.447 |
| *Turdus merula* | 0.02 | 2.394 | 3.263 | 3.310 |
| *Turdus merula* | 0.02 | 2.394 | 3.070 | 3.409 |
| *Turdus merula* | 0.02 | 2.394 | 3.064 | 3.407 |
| *Turdus merula* | 0.02 | 2.394 | 3.100 | 3.361 |
| *Turdus merula* | 1.96 | 2.394 | 3.067 | 3.391 |
| *Turdus merula* | 0.87 | 2.394 | 3.092 | 3.383 |
| *Turdus merula* | 1.75 | 2.394 | 3.068 | 3.373 |
| *Turdus merula* | 0.97 | 2.394 | 3.108 | 3.358 |
| *Turdus philomelos* | 68.05 | 2.370 | 3.073 | 3.324 |
| *Turdus philomelos* | 27.57 | 2.370 | 3.073 | 3.329 |
| *Turdus philomelos* | 7.54 | 2.370 | 3.094 | 3.392 |
| *Turdus philomelos* | 10.89 | 2.370 | 3.111 | 3.306 |
| *Turdus philomelos* | 11.77 | 2.370 | 3.123 | 3.387 |
| *Turdus philomelos* | 12.37 | 2.370 | 3.085 | 3.432 |
| *Turdus philomelos* | 0.02 | 2.370 | 3.081 | 3.436 |
| *Turdus philomelos* | 0.02 | 2.370 | 3.099 | 3.391 |
| *Turdus philomelos* | 0.02 | 2.370 | 3.060 | 3.452 |
| *Turdus philomelos* | 0.02 | 2.370 | 3.042 | 3.413 |
| *Turdus philomelos* | 0.02 | 2.370 | 3.081 | 3.349 |
| *Turdus philomelos* | 0.02 | 2.370 | 3.080 | 3.396 |
| *Turdus philomelos* | 0.02 | 2.370 | 3.080 | 3.349 |
| *Turdus philomelos* | 0.02 | 2.370 | 3.071 | 3.470 |
| *Turdus philomelos* | 0.02 | 2.370 | 3.097 | 3.446 |
| *Turdus philomelos* | 0.02 | 2.370 | 3.013 | 3.367 |
| *Turdus philomelos* | 0.02 | 2.370 | 2.951 | 3.428 |
| *Turdus philomelos* | 1.26 | 2.370 | 3.090 | 3.324 |
| *Turdus philomelos* | 3.91 | 2.402 | 3.074 | 3.413 |
| *Turdus philomelos* | 72.01 | 2.402 | 3.099 | 3.412 |
| *Turdus philomelos* | 19.50 | 2.402 | 3.098 | 3.342 |
| *Turdus philomelos* | 7.30 | 2.432 | 3.079 | 3.418 |
| *Turdus philomelos* | 0.02 | 2.557 | 3.058 | 3.480 |
| *Turdus philomelos* | 0.02 | 2.557 | 3.071 | 3.388 |
| *Turdus viscivorus* | 5.09 | 2.392 | 3.068 | 3.382 |
| *Turdus viscivorus* | 62.66 | 2.392 | 3.073 | 3.423 |
| *Turdus viscivorus* | 49.10 | 2.392 | 3.098 | 3.372 |
| *Turdus viscivorus* | 5.79 | 2.392 | 3.058 | 3.397 |
| *Upupa epops* | 1.95 | 2.383 | 3.041 | 3.425 |
| *Upupa epops* | 1.83 | 2.397 | 3.086 | 3.368 |
| *Upupa epops* | 1.83 | 2.397 | 3.097 | 3.388 |
