# Supplementary material for: Chernobyl Birds Have Smaller Brains
Source: PLoS One. 2011 Feb 4;6(2):e16862. doi: 10.1371/journal.pone.0016862 (PMC3033907; doi:10.1371/journal.pone.0016862)
Supplement: Table S3 — Relationship between morphology and radiation nested within species and species and body mass. Denominator degrees of freedom vary because of missing values due to broken feathers. (DOC) [file pone.0016862.s003.doc]

Table S3. Relationship between morphology and radiation nested within species and species and body mass. Denominator degrees of freedom vary because of missing values due to broken feathers.

|  | Sum of squares | d.f. | F | P |
| --- | --- | --- | --- | --- |
| Beak length: | 233 | 1,482 | 0.02 | 0.89 |
| Beak width | 11559 | 1,482 | 0.96 | 0.33 |
| Beak height | 261 | 1,482 | 0.03 | 0.87 |
| Tarsus length | 1980 | 1.482 | 0.25 | 0.62 |
| Keel length | 13765 | 1,482 | 0.80 | 0.37 |
| Wing length | 3.85 | 1,482 | 0.32 | 0.57 |
| Tail length | 8.89 | 1,474 | 0.17 | 0.68 |
| Central tail length | 9.62 | 1,478 | 0.81 | 0.37 |
| Wingspan | 2956 | 1,482 | 0.08 | 0.85 |
