# Supplementary figures and images for: Chernobyl Birds Have Smaller Brains
Source: PLoS One. 2011 Feb 4;6(2):e16862. doi: 10.1371/journal.pone.0016862 (PMC3033907; doi:10.1371/journal.pone.0016862)

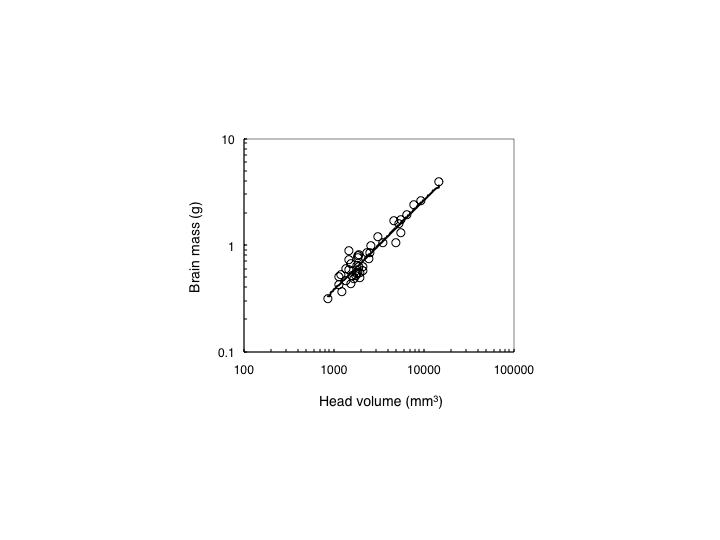

Supplement: Figure S1 — Brain mass (g) in relation to head volume (mm3) in different species of birds. The line is the linear regression line based on log10-transformed variables with the equation log10(Brain mass (g)) = −2.803 + 0.798 log10(Head volume (mm3)), F1,38 = 316.93, r2 = 0.91, P < 0.0001. (TIFF) [file pone.0016862.s004.tiff]
